# Assessment of the Bangla Heart Manual in patients with coronary heart disease and their caregivers in Bangladesh: a feasibility study

**DOI:** 10.1136/bmjopen-2025-102350

**Published:** 2026-03-30

**Authors:** Jamal Uddin, Mithila Faruque, Saidur Rahman Mashreky, Yousra Siddiqueea, Meshkat A Chowdhury, Rezaul Karim, Md Fakhrul Islam Khaled, Louise Taylor, Hasnain M Dalal, Rod Taylor

**Affiliations:** 1 MRC/CSO Social and Public Health Sciences Unit, University of Glasgow, College of Medical Veterinary and Life Sciences, Glasgow, UK; 2 Department of Cardiac Surgery, Physiotherapy Unit, Ibrahim Cardiac Hospital & Research Institute, Dhaka, Bangladesh; 3 Department of Noncommunicable Diseases, Bangladesh University of Health Sciences, Dhaka, Bangladesh; 4 Department of Public Health, North South University, Dhaka, Bangladesh; 5 University Cardiac Centre, Department of Cardiology, Bangabandhu Sheikh Mujib Medical University, Dhaka, Bangladesh; 6 Department of Cardiology, Ibrahim Cardiac Hospital and Research Institute, Dhaka, Bangladesh; 7 The Heart Manual Department, NHS Lothian, Edinburgh, UK; 8 Research, Development and Innovation, Royal Cornwall Hospitals NHS Trust, Truro, UK; 9 Primary Care, University of Exeter Medical School, Truro, UK; 10 MRC/CSO Social and Public Health Sciences Unit & Robertson Centre for Biostatistics, University of Glasgow, Glasgow, UK

**Keywords:** Coronary heart disease, Myocardial infarction, REHABILITATION MEDICINE, Self Care, Exercise

## Abstract

**Objectives:**

To assess the feasibility and acceptability of the home-based Bangla Heart Manual of Cardiac Rehabilitation (CR) programme for people with coronary heart disease (CHD) following revascularisation living in Bangladesh.

**Design and settings:**

Tertiary level cardiac hospital in Dhaka city; a single-centre feasibility pilot study, with a mixed-methods single arm pre–post design.

**Participants:**

The study involved 33 patients with CHD admitted for revascularisation (coronary artery bypass graft or percutaneous coronary intervention) between June and July 2024, selected from 72 screened. Two physiotherapists and one nurse conducted the research, focusing on patients deemed suitable for CR.

**Intervention:**

Selected patients received the Bangla Heart Manual intervention that consisted of a 6-week programme of home-based CR including exercise training, self-care, relaxation, risk factor management and psychological support facilitated by a healthcare professional.

**Primary and secondary outcomes:**

The primary outcomes focused on feasibility, assessing patient recruitment, retention and adherence to the intervention using quantitative and qualitative methods, including interviews with patients, caregivers and healthcare professionals. Secondary outcomes measured patient-reported metrics like health-related quality of life (HeartQoL, EQ-5D-5L), psychological well-being, exercise capacity (Hospital Anxiety and Depression Scale), and serious adverse events (hospitalisation and mortality) before and after the Bangla Heart Manual intervention.

**Results:**

The 33 patients recruited included 29 (88%) males with a mean age of 55 years. The following feasibility outcomes were achieved: 46% (33 patients from 72 screened) recruited, 91% (30/33) retention (complete outcome data at follow-up) and 75% intervention adherence (≥6 sessions attended of 8 sessions). Improvements following CR participation were seen in patient-reported outcomes and exercise capacity. Two deaths and one rehospitalisation occurred during the study.

**Conclusions:**

This study showed that the Bangla Heart Manual home-based CR programme was acceptable and feasible for people with CHD in Bangladesh and healthcare professional staff to deliver. Our results also support the feasibility of recruitment and data collection processes for a future multicentre randomised trial to formally test the clinical and cost effectiveness of the adapted Bangla Heart Manual.

**Trial registration number:**

ISRCTN1545620.

**STRENGTHS AND LIMITATIONS OF THIS STUDY**

- A mixed-method design was employed to collect both quantitative and qualitative data, providing comprehensive assessment study outcomes. Perspectives from patients, caregivers and facilitators, providing a more diverse evaluation of feasibility and acceptability.
- Baseline assessments were conducted before revascularisation, making it difficult to determine whether improvements in exercise capacity and patient-reported outcomes were attributable to cardiac rehabilitation or revascularisation, or both.
- Small sample size and short follow-up period limit the generalisability and interpretability of the findings

## Introduction

Coronary heart disease (CHD) is a single major cause of global disability and death.^1 2^ By 2030, more than 80% of the deaths and disability due to heart diseases across the world will occur in low-income and middle-income countries (LMICs).^3^ CHD is a major socioeconomic burden to LMICs where it directly impacts the people of working age.^4^ Cardiac rehabilitation (CR) is a clinical and cost-effective intervention for people with heart diseases, reducing their risk of recurrent cardiac events and hospital readmissions and improving exercise capacity and health-related quality of life (HRQoL).^5 6^ CR is a multifaceted secondary prevention intervention that involves structured exercise, health education, lifestyle modification and counselling^7^ and is typically delivered by a multidisciplinary team.^5 7 8^ The WHO recommends CR as a priority intervention for secondary prevention and management of heart disease.^9^ However, despite its potential benefits and consistent recommendation in clinical guidelines, CR is globally underused, particularly in LMICs where levels of CHD continue to rise.^10 11^ The traditional model of CR provision of a supervised centre-based programme typically located in a hospital setting poses a number of practical potential access barriers for patients (ie, travelling, time and cost, dislike of group sessions).^12^ The COVID-19 pandemic underlined the need for alternatives to centre-based models of healthcare provision.^13 14^ There is strong evidence that home and centre-based CR programmes yield similar health benefits and have comparable per patient cost.^15^,^17^ The vast majority of the evidence base for CR to date derives from high-income countries. A systematic review and meta-analysis on CR found that 26 trials out of 135 countries (LMICs).^18^ So, caution is needed in directly extrapolating the observed benefits and costs of CR from the high-income settings to LMICs where the nature of healthcare systems in terms of funding sources, access to facilities, staffing capacity and patient income levels is likely to be very different. Despite the availability of advanced cardiac care technology, secondary prevention and CR for heart disease is insufficiently available both in government and private hospitals in Bangladesh. A global survey conducted in 2016–2017 reported only one CR centre-based programme across the whole of Bangladesh.^19 20^ Developing alternative approaches to CR delivery, including home and/or digitally supported models, offers the opportunity to increase access. Using extensive stakeholder participation, our research group has recently developed a culturally adapted version of the ‘Heart Manual’, home-based programme originally developed in the UK, for patients with CHD and their caregivers living in Bangladesh on the ‘Bangla Heart Manual’. Before the Bangla Heart Manual can be used and implemented in routine practice, research is needed to test its feasibility and acceptability.

The objectives of this study were to (1) assess the feasibility and acceptability of delivering the Bangla Heart Manual home-based CR programme for patients with CHD and their caregivers in Bangladesh and (2) explore the change in exercise capacity and patient-reported outcomes following participation in the home-based Bangla Heart Manual programme.

Our hypothesis was that the adapted Bangla Heart Manual was a feasible and acceptable home-based CR programme for people with CHD and their caregivers in Bangladesh.

## Methods and analysis

**Figure 1:**
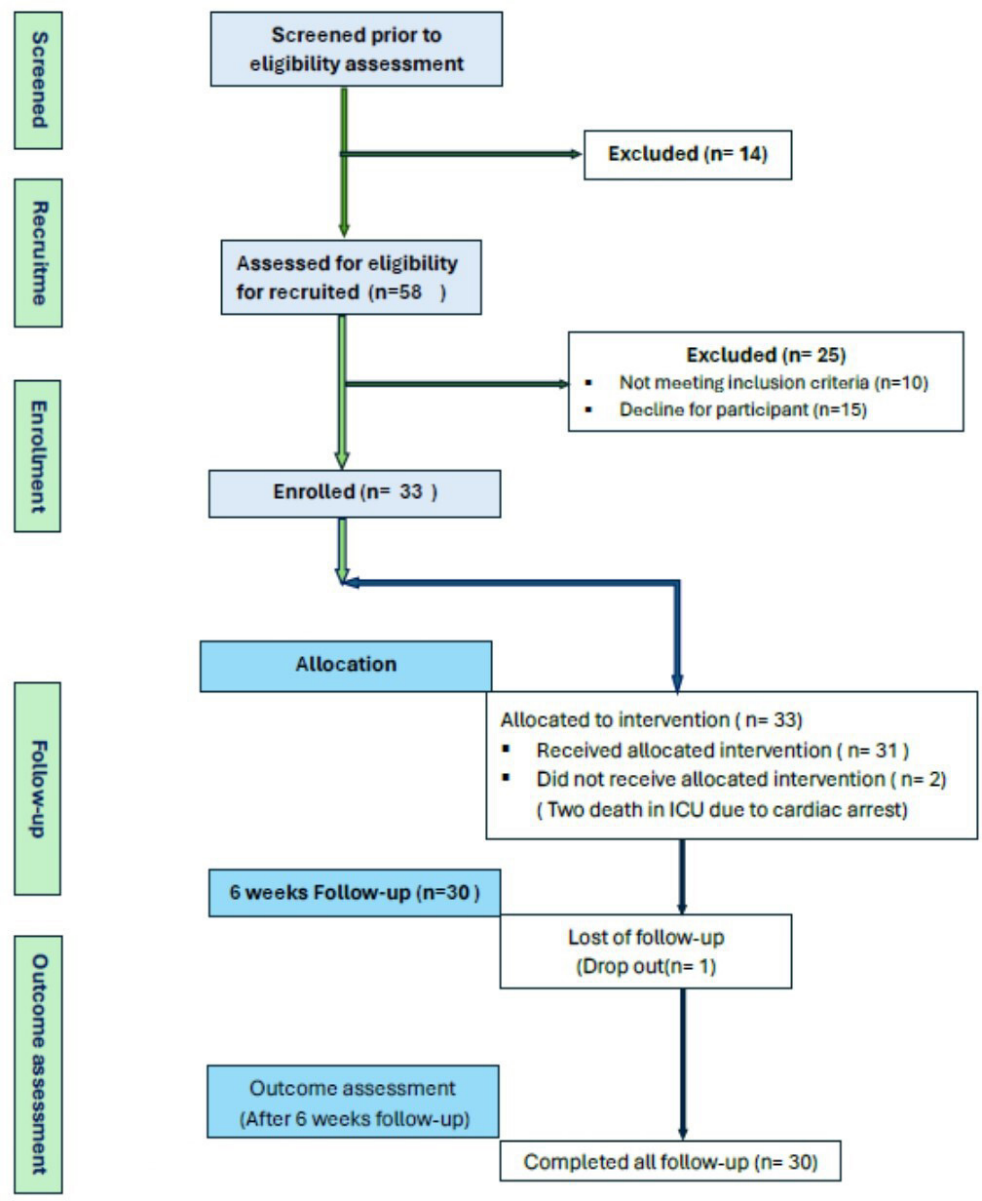
CONSORT diagram for recruitment and enrolment. CONSORT, Consolidated Standards of Reporting Trials.

This feasibility study protocol was pre-registered on the ISRCTN registry: ISRCTN15454620 https://doi.org/10.1186/ISRCTN15454620 (see study protocol as online supplemental file). The study is reported in accordance with the Consolidated Standards of Reporting Trials extension for randomised pilot and feasibility trials (see figure 1).^21^

### Study design

**Figure 2:**
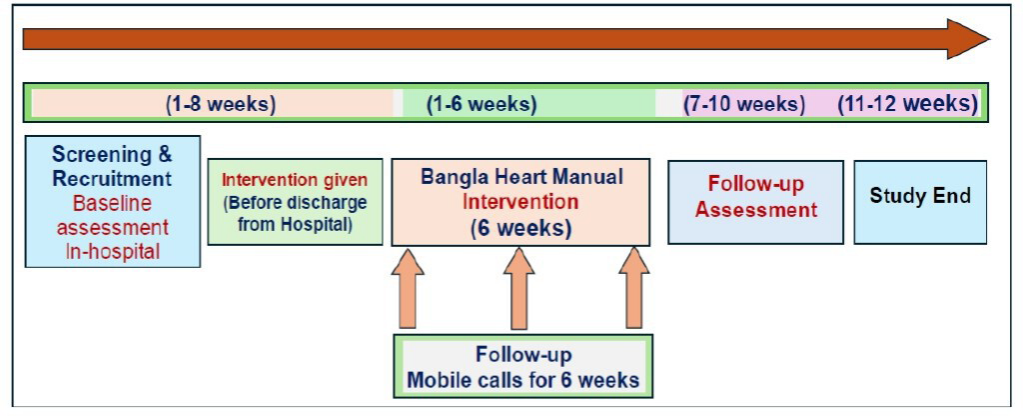
Summary of study participant pathway.

The study employed a mixed-methods single-arm design with pre–post assessment of patient outcomes and qualitative interviews with healthcare staff involved in the intervention delivery, patients and caregivers. Patients were recruited from the Ibrahim Cardiac Hospital & Research Institute based in Dhaka, Bangladesh. Summary of study participant pathway (see figure 2).

### Patient and public involvement

No patients or members of the public were involved in the design, conduct, or reporting of this research.

### Participant identification and recruitment

Consecutive patients aged ≥18 years were recruited with a CHD diagnosis defined as either a previous myocardial infarction (MI) or angina pectoris who were admitted to a hospital for an intended revascularisation intervention (angioplasty and/or coronary artery bypass graft (CABG) procedures) between June and July 2024 and deemed suitable for CR participation by the clinical team. Patients were excluded if they: (1) underwent an emergency revascularisation, (2) had undertaken CR within the last 12 months, (3) had contraindications to exercise testing or exercise training as part of their documented medical history or (4) were unwilling or unable to travel to the clinical site for their research assessments. The schedule of study procedures is shown in online supplemental e-figure 1.

### Bangla Heart Manual intervention

The Bangla Heart Manual is a home-based, health professional-facilitated, 6-week CR programme supporting self-care in patients with CHD. Details of the intervention development process are accepted to publish elsewhere.^22^ The programme is described in detail according to the Template for Intervention Description and Replication checklist (see online supplemental eAppendix, eTable 1). The Bangla Heart Manual intervention includes specific exercise programmes that meet the needs of patients following MI, CABG and percutaneous coronary intervention (PCI).

#### Coronary artery bypass grafting

The exercise programme incorporated sternal precautions for the first 6 weeks, including avoidance of pushing, pulling, trunk rotation and bilateral overhead arm movements. Bed mobility used the log roll technique during weeks 1–2. Upper limb exercises (eg, shoulder shrugs, flexion and abduction) were initiated in weeks 1–2 with movements restricted to ≤shoulder height. Progression was conservative to protect sternal healing.

#### Percutaneous coronary intervention

Patients resumed light daily activities during Week 1, with walking initiated at 5–10 min, 2–3 times/day. From week 2 onward, walking duration increased to 20–30 min at moderate intensity. Light resistance training (eg, squats, biceps curls) and gentle stretching were introduced after week 2. No sternal precautions were required.

Healthcare professionals, including physicians, physiotherapists, nurses and dietitians, were trained to deliver these tailored components appropriately. In brief, the Bangla Heart Manual includes the key elements of a comprehensive CR programme, that is, exercise training, psychological support and education around self-management including medical therapy and healthy lifestyle. Patients were introduced and educated to the patients and caregivers to the intervention materials by a physiotherapist during the hospital stay. On discharge, patients then received a weekly phone call to discuss their progress over 6 weeks with the final contact again taking place in the hospital setting. The seven health professionals (three physiotherapists, two nurses, two medical technologists) with CR experience attended the 2 days of online training delivered by the staff of the Heart Manual Office in the UK. Training focused on the seven steps of successful facilitation of the Bangla Heart Manual: (1) building rapport; (2) assessing the needs and building an understanding of patients with CHD; (3) support self-management and progress monitoring; (4) discussing 6 weeks exercise and well-being; trained how to measure ISWT; (5) summarise and plan next steps; (6) reviewing progress and (7) supporting long-term maintenance. All study participants continued to receive their usual medical management and care according to local hospital and national CHD guidelines.

### Data collection procedure

Baseline and follow-up data collection were conducted by a trained researcher at two time points—baseline: when the patient was in hospital for percutaneous event (before CR intervention) and follow-up: follow-up visit following the 6 weeks of CR intervention. Baseline data included baseline medical and demographic characteristics and at both baseline and 3-month follow-up: exercise capacity, a range of participant-reported questionnaires (see below) and patient and caregiver satisfaction. Data were obtained via face-to-face interview and from medical records. Semistructured interviews with patients and clinicians were completed by phone by a trained research interviewer. A standardised interview guide was used for all semistructured interview qualitative interviews.^23^ Patient interview guides included open-ended questions about patients’ transition into home care, their experience with Bangla Heart Manual via phone call and their current lifestyle and follow-up plan. Clinician interview guides included open-ended questions about clinicians’ perception of the programme, experience working with patients and barriers to implementation. All interviews were audio recorded and professionally transcribed. Patient and caregiver treatment satisfaction level will be assessed by using this questionnaire (see online supplemental e-figure 2).

### Outcomes measures

#### Primary outcomes

The primary outcomes were the feasibility and the acceptability of the adapted Bangla Heart Manual CR intervention. There were three prespecified primary outcome criteria: study recruitment (number of consented participants relative to the total number of eligible patients screened), study retention (proportion of study participants who completed outcomes at 3-month follow-up), adherence to the intervention (proportion of completion of the Bangla Heart Manual intervention components according to training logs where ≥6 of 8 sessions attended seemed adequate), (see table 1). These criteria informed potential progression to a potential future full multicentre randomised controlled trial to assess the clinical and cost effectiveness of Bangla Heart Manual.

**Table 1:** Summary of feasibility outcomes

Outcomes	Definition	Prespecified success criteria	Study outcome
Recruitment	Number of consented participants relative to the total number of eligible patients approached for consent	Recruitment rate of ≥30%	33 patients were recruited/72 were screened over a 2- month period (33/2) ≥16 patients per month, 46%
Retention	Number of participants who complete the final study assessment relative to the total number of participants enrolled for pilot trial.	Retention rate of ≥80%	2 deaths and dropout 1 (30/33), 91%
Adherence	Percentage of completion of the Bangla Heart Manual intervention components according to training logs.	Intervention adherence of ≥75%	(6 sessions out of 8 sessions delivered in 6 weeks programme), 75%

Patients and their caregivers’ satisfaction with receiving the Bangla Heart Manual was assessed using a level by five-grade Likert scale at 3-month follow-up^24 25^ (see online supplemental eAppendix, eTable 2).

#### Secondary outcomes

Secondary outcome measures by time point (see online supplemental eAppendix, eTable 3). Secondary outcomes included participant exercise capacity (Incremental Shuttle Walk Test (ISWT)^26^ and patient-reported outcomes (disease-specific HRQoL—Bangla HeartQoL,^27^ generic HRQoL—EuroQoL/EQ-5D-5L^28^ and psychological well-being—Hospital Anxiety and Depression Scale (HADS),^29^ assessed at baseline and 3-month follow-up.

### Data analysis

To achieve the objectives of this study and based on the recommendation of Treweek *et al*^30^ for pilot and feasibility trials of a minimum of 10–15 participants, the study planned to recruit a total of 30 patients over a 2-month period allowing for loss to follow-up of up to 20% at 3 months follow-up. Preintervention and postintervention participant outcome data are presented descriptively as frequencies (and percentages) for binary outcomes, and as mean (SD) for continuous outcomes. Pre–post differences in outcomes are presented as means and 95% CIs. Given the exploratory nature of the study, the focus of outcome interpretation was around 95% CIs rather than p values. Quantitative data were analysed using SPSS V.23.0 with the significance level set at p<0.05. Baseline data were presented for continuous variables as means and SD, while for categorical variables, frequencies and percentages were used to represent the data. Pre–post differences in outcomes are presented as means and 95% CIs and calculated using paired-sample t-tests. P-values are reported for descriptive purposes only. Interview data (qualitative data) were analysed using thematic analysis, as described by Braun and Clarke (2006),^31^ by two qualitative researchers (JU and YS) who developed codes and identified themes.

## Results

A detailed summary of the baseline sociodemographic, comorbidities, clinical and biomedical characteristics of the 33 study participants. The data show a predominantly male sample (88%, n=29) with a mean age of 55 years (SD 7.7). Clinically, the majority of participants underwent CABG (70%, n=23) compared with PCI (30%, n=10). A high prevalence of comorbidities was observed, including diabetes (85%, n=28) and dyslipidaemia (82%, n=27). This baseline characterisation is crucial for understanding the population to whom the feasibility results apply and for comparing with future study populations (see table 2).

**Table 2:** Participants’ demographic, comorbidities, clinical and biomedical characteristics

Gender	
Male	29 (88%)
Age, years	55±7.7
BMI, kg/m^2^	25.4 (2.6)
Education	
Primary school completed	3 (12%)
High school completed	12 (36%)
Higher secondary school and graduation completed	12 (36%)
Post graduation completed	6 (18%)
Marital status	
Married	33 (100%)
Intervention procedure	
CABG	23 (70%)
PCI	10 (30%)
Comorbidities	
Diabetes	28 (85%)
Dyslipidaemia	27 (82%)
CKD	3 (9%)
Stroke	2 (6%)
COPD	1 (3%)
Arthritis	0 (0%)
Risk factors	
Smoker	19 (58%)
Physically inactive	17 (52%)
Fatty food	20 (61%)
Family history	15 (45%)
Alcoholic	0 (0%)
Biomedical parameters	
Total cholesterol	138.8 (42.3) mg/dL
Triglyceride	163.3 (81.7) mg/dL
LDL	66.5 (30.5) mg/dL
HDL	39.6 (8.1) mg/dL
Clinical parameters	
LVEF (%)	52 (0.09)

BMI, body mass index; CABG, coronary artery bypass grafting; CKD, chronic kidney disease; COPD, chronic obstructive pulmonary disease; HDL, high-density lipoproteins; LDL, low-density lipoproteins; LVEF, left ventricular ejection fraction; PCI, percutaneous coronary intervention.

### Primary feasibility outcomes

This study outlines the primary feasibility outcomes against predefined success criteria. It confirms that all three primary feasibility benchmarks were met: a recruitment rate of 46% (33 of 72 screened) exceeded the prespecified target of ≥30%; a retention rate of 91% (30 of 33 completed follow-up) surpassed the ≥80% target; and intervention adherence was 75%, meeting the ≥75% criterion. These findings provide strong evidence for the feasibility of conducting a larger trial and are central to the study’s main objective (see table 1).

Some patients did not consent to participate due to lack of confidence in completing the study, their caregivers were not interested, or they felt under too much stress with the process of revascularisation. Intervention adherence was 75% (Completion of the Bangla Heart Manual intervention components according to training logs) (see table 1). 30 of 33 (91%) participants provided full outcome data at 3-month follow-up. Two patients died (one due to septic shock and one with cardiac arrest after CABG) and one patient dropped out. Neither of these two serious adverse events was deemed related to the CR intervention.

Patients and caregivers reported a high level of satisfaction with study participation and the CR intervention (see table 3).

**Table 3:** Level of satisfaction of patients and caregivers assessed at 3-month F/U

	Mean (SD) and 95% CI
Patients	4.3 (0.6)
Caregivers	4.4 (0.6)

F/U, follow-up.

This study reports the level of satisfaction with the Bangla Heart Manual programme among patients and their caregivers at the 3-month follow-up, measured on a 5-point Likert scale. The high mean satisfaction scores for both patients (4.3, SD 0.6) and caregivers (4.4, SD 0.6) indicate that the intervention was highly acceptable to the end-users, supporting its potential for real-world implementation (see table 3).

### Secondary outcomes

Improvements at 3-month follow-up compared with baseline were seen in exercise capacity assessed by ISWT (see table 4).

**Table 4:** Secondary outcomes: exercise capacity (ISWT) at baseline and 3-month F/U

	ISWT (metres) mean (SD)	Pre-post ISWT (metres) mean difference (95% CI)	P value
Baseline (n=17)	91.2 (33.3)	62.2 (36.27 to 88.18)	<0.0001
Follow-up (n=19)	155.3 (33.1)		

P values derived from exploratory paired-sample t-tests.

F/U, follow-up; ISWT, Incremental Shuttle Walk Test.

This study presents the changes in exercise capacity, measured by the ISWT, from baseline to the 3-month follow-up. The analysis shows a significant and clinically meaningful mean improvement of 62.2 m (95% CI 36.27 to 88.18, p<0.0001), increasing from 91.2 m (SD 33.3) at baseline to 155.3 m (SD 33.1) at follow-up. While exploratory, this positive signal supports the potential efficacy of the intervention (see table 4).

This feasibility study offers a comprehensive overview of changes in all secondary patient-reported outcomes from baseline to the 3-month follow-up. It details significant improvements across multiple domains: disease-specific quality of life (HeartQoL physical and emotional subscales), psychological well-being (anxiety and depression scores on the HADS), and generic health status (all five dimensions of the EuroQol 5-Dimension 5-Level (EQ-5D-5L), the EuroQol Visual Analogue Scale (EQ-VAS) and the EQ-5D-5L index score using Indian value norms). Patient-reported outcomes of the HeartQoL, HADS and EQ-5D-5L (see table 5). These consistent positive findings across diverse measures reinforce the potential holistic benefit of the Bangla Heart Manual.

**Table 5:** Secondary outcomes: HeartQoL physical and emotional subscale, HADS and EQ-5D-5L at baseline and 3-month follow-up

HeartQoL physical and emotional subscale				
	Baseline (n=33), mean (SD)	Follow-up (n=30), mean (SD)	Mean difference and 95% CI	P value
Physical subscale	1.69 (0.64)	2.62 (0.41)	0.94 (0.66 to 1.21)	<0.0001
Emotional subscale	1.78 (0.79)	2.67 (0.55)	0.87 (0.47 to 1.27)	

HADS				
	Baseline (n=33), mean (SD)	Follow-up (n=30), mean (SD)	Mean difference and 95% CI	P value
Anxiety	6.79 (4.08)	1.55 (1.56)	5.24 (3.94 to 6.55)	<0.0001
Depression	5.27 (3.39)	2.12 (2.30)	1.93 (1.14 to 2.72)	

EQ-5D-5L dimensions				
	Baseline (n=30), mean (SD)	Follow-up (n=29), Mean (SD)	Mean difference and 95% CI	P value
Mobility	2.23 (1.15)	1.13 (0.35)	1.10 (0.64 to 1.56)	<0.0001
Self-care	1.74 (1.06)	1.2 (0.41)	0.52 (0.15 to 0.92)	0.01
Usual activities	1.84 (1.07)	1.1 (0.31)	0.76 (0.34 to 1.17)	0.0008
Pain or discomfort	2.19 (0.91)	1.5 (0.51)	0.66 (0.23 to 1.07)	0.004
Anxiety or depression	2.29 (0.86)	1.3 (0.47)	1.03 (0.69 to 1.38)	<0.0001

EQ-5D-5L VAS				
EQ-VAS	Baseline (n=30), mean (SD)	Follow-up (n=29), mean (SD)	Mean difference and 95% CI	P value
VAS score, mean (SD)	68 (18)	86 (09)	17 (11.16 to 22.91)	<0.0001

EQ-5D-5L utility scores using Indian population health value norms				
EQ-5D-5L pre and post (value norms)	Baseline (n=30), mean (SD)	Follow-up (n=29), mean (SD)	Mean difference and 95% CI	P value
EQ-5D-5L index score	0.69 (0.37)	0.94 (0.06)	0.25 (0.11 to 0.39)	0.00094

P values derived from exploratory paired-sample t-tests.

EQ-5D-5L, EuroQol 5-Dimension 5-Level; HADS, Hospital Anxiety and Depression Scale; HeartQoL, health-related quality of life; VAS, Visual Analogue Scale.

### Patient interviews

In this study, table 6 presents illustrative qualitative data from semi-structured interviews with patients and clinicians. For patients, the themes of ‘Knowledge and practice of home CR’ and ‘Self-motivation and follow-up plan’ are exemplified by quotes highlighting their learning and positive behavioural changes. For clinicians, the themes of a ‘Systematic and comprehensive approach’ and ‘Motivation and involved clinical practice’ are supported by quotes expressing their satisfaction with the programme’s structure and its potential for integration into routine care. This qualitative evidence provides crucial context and depth, explaining the high satisfaction and adherence rates observed in the quantitative data.

**Table 6:** Examples of quotes to illustrate patient and clinician (facilitators) themes

Theme	%, participants	Quotes
Patients**:** Knowledge and practice of home CR Self-motivation and follow-up plan	N=6 86% 75%	“We learned about the coronary heart disease and its treatment option”. “We did some exercise and not in bed diet but good to know the importance of self-management, diet and relaxation techniques”.
Clinicians: Systematic and comprehensive approach to home CR Motivation and involved clinical practice	N=3 91% 82%	“I liked the way intervention delivered sequentially by giving first visit, second visit etc and their contents”. “Generally, patients are excited about the programme. Involvement of caregivers is a great idea” “they were highly motivated to keep continue this experience into their clinical practice”.

CR, cardiac rehabilitation.

We developed a set of nine codes that were applied to the transcripts to identify two overarching themes that emerged (see table 6) : Theme 1: Knowledge and practice of home CR: Patients get some knowledge regarding heart disease and its management. One patient said that “ learn about the coronary heart disease and its treatment option”. Other patients explain that “We did some exercise and not in bed diet but good to know the importance of self-management diet and relaxation”. Patients reported they had a very positive experience of effective communication from the physiotherapist at the hospital, home (via phone call) and across all of the parts of the Bangla Heart Manual programme.

Theme 2: self-motivation to change lifestyle and follow-up plan: Patients were motivated to make lifestyle changes. Patients expressed that they followed the healthy diet (following the diet chart book provided) and followed the instruction to avoid fatty and junk food. Most of the patients commented that they were motivated to regain prior functioning by doing proper weekly exercise and walking programmes. One patient states that “ they workout properly the weekly home exercise and relaxation practice that has given in to the book and practically treated by the physiotherapist the animated and audiovisual version of exercise and relaxation section”. One patient stated that “the clinicians were instrumental in helping them to achieve these goals”. Another patient stated that “the physiotherapist was motivating with having weekly phone call that’s really encouraging”.

### Clinician interviews

We developed seven codes applied to the transcripts to identify themes. Two themes emerged (see table 6).

Theme 1: Systematic and comprehensive approach to home CR delivery: Clinicians described the home-based CR programme as a systematic approach to complete rehabilitation of patients at their home and emphasising the importance of patients having access to the programme materials. One facilitator stated, “ I liked the way intervention delivered sequentially by giving first visit, second visit etc and their contents”. Another facilitator explained that “ by the time I discharged the patient they really get clear demonstration of exercise, educated to diet management, for stress and mental health management practiced relaxation, educate on risk factor management which really ties into managing their disease”.

Theme 2: Motivation and involved clinical practice: Clinicians spoke about their experiences of the challenges with working with patients who were not motivated to change or struggled with the components of the educational part of the programme and the importance of engaging patients caregivers. Facilitators expressed that “they were highly motivated to keep continue this experience into their clinical practice”.

## Discussion

This study aimed to determine the feasibility and acceptability of the Bangla Heart Manual, an adapted 6-week home-based CR intervention, for people with CHD in Bangladesh in order to inform both the potential future clinical access to the intervention and also feasibility of undertaking a future definitive randomised controlled trial to assess its clinical and cost-effectiveness. Our mixed methods analysis provides supportive evidence of feasibility and acceptability of the intervention with patients and healthcare staff delivering the intervention. Good levels of intervention adherence (75% attended a minimum of 80% of intervention sessions) were seen and patients and caregivers reported high levels of satisfaction following their participation in the 6-week facilitated period of intervention delivery. The recruitment rate was 46% (success rate of recruitment is ≥30%), which was shown to be acceptable and outcome retention was 91% participants (30/33) completing outcomes at 3-month follow-up. Although not formally powered for efficacy, this study also showed improvements in exercise capacity and patient-reported outcomes of HRQoL and psychological well-being following participation in the CR intervention. Finally, the Bangla Heart Manual appeared to be a safe intervention for patients with CHD following revascularisation. Although the evidence base for CR in the LMIC setting is relatively limited and continues to evolve, our results appear to be consistent with the benefits of CR reported in other studies in low resource settings. A single-centre randomised trial comparing home-based CR plus usual care versus usual care alone in Dhaka, Bangladesh, showed benefits in patient-reported outcomes of mental well-being and exercise capacity with CR. This study provides proof of concept of the feasibility and acceptability of a home-based CR approach in this setting.^32^

The Yoga-CaRe multicentre randomised trial in India reported improved self-rated health and return to preinfarct activities after acute MI with a yoga-based rehabilitation programme.^33^ Recent studies in Bangladesh and India show improved HRQoL of post-PCI and CABG, with greater physical than emotional gains.^34^ CABG patients had higher HRQoL than PCI patients at 6–24 months.^34^ Both CABG and PTCA enhance HRQoL,^35^,^38^ but CABG improves both physical and psychological domains, while PTCA mainly benefits physical health.^39^ Indian studies found significant physical but limited psychological HRQoL improvements post-revascularisation.^40 41^ These findings highlight the varying impacts of revascularisation on HRQoL.

To our knowledge, this is the first study to assess the feasibility and acceptability of delivering a home-based CR programme in Bangladesh. A home-based model of delivery offer offers an affordable and scalable approach to improve CR in the wider LMIC setting. This study provides proof-of-concept for a culturally adapted, home-based CR model delivered. However, this study has some limitations. First, the single-centre recruitment from an urban tertiary hospital and the predominantly male sample limit the generalisability of our feasibility findings to the wider Bangladeshi population, including women and those in rural or primary care settings. Second, the lack of a control group precludes attribution of observed improvements solely to the CR intervention, as these may partly reflect natural recovery following revascularisation. In addition, the small sample size and short follow-up period preclude definitive conclusions regarding efficacy. Additionally, the reliance on self-reported measures for HRQoL and psychological well-being may be subject to reporting bias. Finally, we acknowledge that we were not able to support the inclusion of patient and public involvement in this study.

### Implications for practice and future research

Worldwide, CR programme uptake attendance rate has been suboptimal. In 2019, in the UK and USA, only 50% of all patients who were eligible attended the CR programme.^42 43^ A noticeable change in the healthcare services provided has been seen in response to the COVID-19 pandemic. Between 2018 and 2021, in the UK, the proportion of home-based CR programmes rose from 16% to 70%.^42^ According to the ICCPR survey, COVID-19 has impacted CR delivery around the globe, resulting in at least temporary cessation of ~75% of CR programmes.^44 45^ The availability of alternative modes of delivery to traditional centre-based CR programmes, including home and digitally supported models, is key to improving global CR access. Our study supports the potential to increase access to CR in Bangladesh using the Bangla Heart Manual, a model of home-based CR supported by healthcare professionals using a mobile phone. The results of this study indicate the importance and feasibility of a larger randomised controlled trial to formally assess the clinical and cost-effectiveness of Bangla Heart Manual for patients with CHD and their caregivers in Bangladesh.

## Conclusions

The culturally adapted Bangla Heart Manual was found to be a feasible and acceptable home-based CR intervention for Bangladeshi patients with CHD and their caregivers and for healthcare professionals to deliver. While not formally powered for efficacy, the observed improvements in patient exercise capacity, HRQoL and psychological well-being following CR participation are supportive of the value of this intervention. A fully-powered multicentre randomised trial is needed to confirm the clinical and cost effectiveness of Bangla Heart Manual for patients with CHD following revascularisation in Bangladesh.

## Supplementary material

10.1136/bmjopen-2025-102350 online supplemental file 1

10.1136/bmjopen-2025-102350 online supplemental file 2

10.1136/bmjopen-2025-102350 online supplemental file 3

10.1136/bmjopen-2025-102350 online supplemental file 4

10.1136/bmjopen-2025-102350 online supplemental file 5

10.1136/bmjopen-2025-102350 online supplemental file 6

## Data Availability

Data are available on reasonable request. All data relevant to the study are included in the article or uploaded as supplementary information.

## References

[1] Roth GA, Abate D, Abate KH (2018). Global, regional, and national age-sex-specific mortality for 282 causes of death in 195 countries and territories, 1980–2017: a systematic analysis for the Global Burden of Disease Study 2017. The Lancet.

[2] Khan MA, Hashim MJ, Mustafa H (2020). Global Epidemiology of Ischemic Heart Disease: Results from the Global Burden of Disease Study. Cureus.

[3] Fernandes JC (2018). Global, regional, and national disability-adjusted life-years (DALYs) for 359 diseases and injuries and healthy life expectancy (HALE) for 195 countries and territories, 1990-2017: a systematic analysis for the Global Burden of Disease Study 2017. Lancet.

[4] Ramaraj R, Chellappa P (2008). Cardiovascular risk in South Asians. Postgrad Med J.

[5] Grace SL, Turk-Adawi KI, Contractor A (2016). Cardiac rehabilitation delivery model for low-resource settings. Heart.

[6] Anderson L, Thompson DR, Oldridge N (2016). Exercise-based cardiac rehabilitation for coronary heart disease. Cochrane Database Syst Rev.

[7] Taylor RS, Sagar VA, Davies EJ (2014). Exercise-based rehabilitation for heart failure. Cochrane Database Syst Rev.

[8] Taylor RS, Walker S, Smart NA (2019). Impact of exercise-based rehabilitation in patients with heart failure (ExTraMATCH II) on exercise capacity and health-related quality of life: a meta-analysis of individual participant data from randomised trials. J Am Coll Cardiol.

[9] Chestnov O (2013). World Health Organization global action plan for the prevention and control of noncommunicable diseases.

[10] Turk-Adawi K, Sarrafzadegan N, Grace SL (2014). Global availability of cardiac rehabilitation. Nat Rev Cardiol.

[11] Ragupathi L, Stribling J, Yakunina Y (2017). Availability, Use, and Barriers to Cardiac Rehabilitation in LMIC. Glob Heart.

[12] Ruano-Ravina A, Pena-Gil C, Abu-Assi E (2016). Participation and adherence to cardiac rehabilitation programs. A systematic review. Int J Cardiol.

[13] Taylor RS, Hayward C, Eyre V (2015). Clinical effectiveness and cost-effectiveness of the Rehabilitation Enablement in Chronic Heart Failure (REACH-HF) facilitated self-care rehabilitation intervention in heart failure patients and caregivers: rationale and protocol for a multicentre randomised controlled trial. BMJ Open.

[14] Thomas E, Gallagher R, Grace SL (2021). Future-proofing cardiac rehabilitation: Transitioning services to telehealth during COVID-19. Eur J Prev Cardiol.

[15] McDonagh ST, Dalal H, Moore S (2023). Home-based versus centre-based cardiac rehabilitation. Cochrane Database Syst Rev.

[16] Dalal HM, Zawada A, Jolly K (2010). Home based versus centre based cardiac rehabilitation: Cochrane systematic review and meta-analysis. BMJ.

[17] Jolly K, Taylor R, Lip GY (2007). The Birmingham Rehabilitation Uptake Maximisation Study (BRUM). Home-based compared with hospital-based cardiac rehabilitation in a multi-ethnic population: cost-effectiveness and patient adherence. Health Technol Assess.

[18] Mamataz T, Uddin J, Ibn Alam S (2022). Effects of cardiac rehabilitation in low-and middle-income countries: A systematic review and meta-analysis of randomised controlled trials. Prog Cardiovasc Dis.

[19] Supervia M, Turk-Adawi K, Lopez-Jimenez F (2019). Nature of Cardiac Rehabilitation Around the Globe. EClinicalMedicine.

[20] Alam SI, Uddin J, Khaled FI (2021). Secondary Preventive Care for Cardiovascular Diseases in Bangladesh: A National Survey. Glob Heart.

[21] Eldridge SM, Chan CL, Campbell MJ (2016). CONSORT 2010 statement: extension to randomised pilot and feasibility trials. BMJ.

[22] Uddin J, Faruque M, Mashreky SR (2026). Adaptation of an evidence-based home cardiac rehabilitation programme for people with coronary heart disease in Bangladesh. BMC Health Serv Res.

[23] Kallio H, Pietilä AM, Johnson M (2016). Systematic methodological review: developing a framework for a qualitative semi-structured interview guide. J Adv Nurs.

[24] Lorish CD, Maisiak R (1986). The face scale: A brief, nonverbal method for assessing patient mood. Arthritis Rheum.

[25] Yoshihara S, Kanno N, Fukuda H (2015). Caregiver treatment satisfaction is improved together with children’s asthma control: Prospective study for budesonide monotherapy in school-aged children with uncontrolled asthma symptoms. Allergol Int.

[26] Singh SJ, Jones PW, Evans R (2008). Minimum clinically important improvement for the incremental shuttle walking test. Thorax.

[27] Oldridge N, Höfer S, McGee H (2014). The HeartQoL: Part I. Development of a new core health-related quality of life questionnaire for patients with ischemic heart disease. Eur J Prev Cardiol.

[28] Rabin R, de Charro F (2001). EQ-5D: a measure of health status from the EuroQol Group. Ann Med.

[29] Zigmond AS, Snaith RP (1983). The hospital anxiety and depression scale. Acta Psychiatr Scand.

[30] Treweek S (2015). Complex interventions in health.

[31] Braun V, Clarke V (2006). Using thematic analysis in psychology. Qual Res Psychol.

[32] Uddin J, Joshi VL, Moniruzzaman M (2020). Effect of Home-Based Cardiac Rehabilitation in a Lower-Middle Income Country: Results from a Controlled Trial. J Cardiopulm Rehabil Prev.

[33] Prabhakaran D, Chandrasekaran AM, Singh K (2020). Yoga-Based Cardiac Rehabilitation After Acute Myocardial Infarction: A Randomized Trial. J Am Coll Cardiol.

[34] Rahman A, Haider R, Shirin H (2024). Evaluate the Quality of Life in Patients With Percutaneous Coronary Intervention Versus Coronary Artery Bypass Graft. Cureus.

[35] Dunning J, Waller JRL, Smith B (2008). Coronary Artery Bypass Grafting is Associated With Excellent Long-Term Survival and Quality of Life: A Prospective Cohort Study. Ann Thorac Surg.

[36] Rumsfeld JS, Magid DJ, Plomondon ME (2003). Health-related quality of life after percutaneous coronary intervention versus coronary bypass surgery in high-risk patients with medically refractory ischemia. J Am Coll Cardiol.

[37] Kattainen E, Meriläinen P, Sintonen H (2006). Sense of coherence and health-related quality of life among patients undergoing coronary artery bypass grafting or angioplasty. Eur J Cardiovasc Nurs.

[38] Favarato ME, Hueb W, Boden WE (2007). Quality of life in patients with symptomatic multivessel coronary artery disease: a comparative post hoc analyses of medical, angioplasty or surgical strategies-MASS II trial. Int J Cardiol.

[39] Chaudhury S, Sharma S, Pawar AA (2006). Psychological Correlates of Outcome after Coronary Artery Bypass Graft. Med J Armed Forces India.

[40] Cohen DJ, Van Hout B, Serruys PW (2011). Quality of life after PCI with drug-eluting stents or coronary-artery bypass surgery. N Engl J Med.

[41] Chaudhury S, Srivastava K (2013). Relation of Depression, Anxiety, and Quality of Life with Outcome after Percutaneous Transluminal Coronary Angioplasty. ScientificWorldJournal.

[42] Singh S, Sinha VK, Singh S (2020). Quality of life after coronary artery bypass graft & percutaneous transluminal coronary angioplasty. Indian J Med Res.

[43] British Heart Foundation (2019). The national audit of cardiac rehabilitation (NACR) quality and outcomes report. https://www.bhf.org.uk.

[44] Ades PA, Khadanga S, Savage PD (2022). Enhancing participation in cardiac rehabilitation: Focus on underserved populations. Prog Cardiovasc Dis.

[45] Ghisi GL de M, Xu Z, Liu X (2021). Impacts of the COVID-19 Pandemic on Cardiac Rehabilitation Delivery around the World. gh.

