# Supplementary material for: Assessment of the Bangla Heart Manual in patients with coronary heart disease and their caregivers in Bangladesh: a feasibility study
Source: BMJ Open. 2026 Mar 30;16(3):e102350. doi: 10.1136/bmjopen-2025-102350 (PMC13052692; doi:10.1136/bmjopen-2025-102350)
Supplement: online supplemental file 1 [file bmjopen-16-3-s001.pdf]

	Feasibility Study period and visit								
	Enrolment	***Baseline (in-hospital)***	***6 weeks (each week mobile call) follow-up***						***Final follow-up outcome***
Time point	***-t_1***	***T1***	***Wk-1***	***Wk-2***	***Wk-3***	***Wk-4***	***Wk-5***	***Wk-6***	***T2***
Enrolment	×								
Eligibility screen *(Inclusion/exclusion criteria)*	×								
Informed consent	×	×							
Allocation		×							
Intervention		↔	↔	↔	↔	↔	↔	↔	
Baseline assessment (demographic and clinical variables from hospital record files)	×	×							
Exercise capacity and patient reported outcomes. (pre and post procedure, In hospital and follow-up by mobile call)									
✓ *Incremental shuttle walk test*		×							×
✓ *Bangla HeartQoL questionnaire*		×							×
✓ *EQ-5D-5L questionnaire*		×							×
✓ *Hospital Anxiety and Depression*		×							×
✓ *Scale (HADS) questionnaire*		×							×
✓ *Serious adverse events (hospitalization/ death)*		×	↔	↔	↔	↔	↔	↔	×
✓ *Patients and their caregivers satisfaction*									×

eFigure 1: Schedule of study procedures:
