# Supplementary material for: Assessment of the Bangla Heart Manual in patients with coronary heart disease and their caregivers in Bangladesh: a feasibility study
Source: BMJ Open. 2026 Mar 30;16(3):e102350. doi: 10.1136/bmjopen-2025-102350 (PMC13052692; doi:10.1136/bmjopen-2025-102350)
Supplement: online supplemental file 2 [file bmjopen-16-3-s002.pdf]

e-Figure 2. Patient and caregiver treatment satisfaction questionnaire.

Question: How satisfied are (patients with coronary heart disease) after CABG and or PCI received home based Cardiac rehabilitation intervention with weekly mobile call for 6 weeks?

Please chose the option that most closely matches your response.

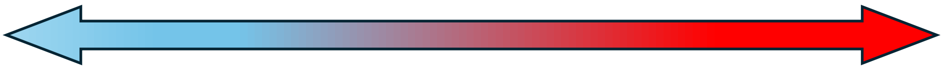

Extremely satisfied (1) **Extremely dissatisfied (5)**

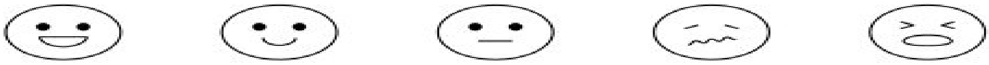

**Extremely satisfied**	**Satisfied**	**Neither satisfied nor dissatisfied**	**Dissatisfied**	**Extremely dissatisfied**
**5**	**4**	**3**	**2**	**1**

The face scale has five grades [1=extremely dissatisfied) to 5=extremely satisfied].
