# Supplementary material for: Assessment of the Bangla Heart Manual in patients with coronary heart disease and their caregivers in Bangladesh: a feasibility study
Source: BMJ Open. 2026 Mar 30;16(3):e102350. doi: 10.1136/bmjopen-2025-102350 (PMC13052692; doi:10.1136/bmjopen-2025-102350)
Supplement: online supplemental file 3 [file bmjopen-16-3-s003.pdf]

e Table 1. Description of Bangla Heart Manual intervention according to the TIDieR checklist:

Item			Item description
1.	Brief Name	Provide the name or a phrase that describes the intervention	**'The Bangla Heart Manual'** intervention for patients with MI and revascularization
2.	Why	Describe any rationale, theory, or goal of the elements essential to the intervention	The 'Heart Manual' is designed to guide the patient and (their) family caregivers to use a six-week home-based CR programme consisting of written materials, sections to record progress, a Q&A audio, and relaxation programme facilitated by a specially trained physiotherapist. The 'Bangla Heart Manual' not only focuses on short-term recovery but aims to assist the patient in adopting and maintaining self-management skills, promoting the maintenance of well-being from their event or intervention to long-term self-management. It also contains all the elements of a comprehensive rehabilitation programme: Health behaviour change and education, lifestyle risk management, exercise, medical risk management and psychological health and wellbeing.<br>Surveillance report 2017, National Institute for Health and Care Excellence (NICE) has identified the Heart Manual as a comprehensive, validated home-based cardiac rehabilitation programme in its clinical guidelines for secondary prevention since 2007. [50]<br>Robust evidence found in a different systematic review, the home-based programmes using the Heart Manual were as equally effective as hospital-based rehabilitation. [51] It also concluded that the Heart Manual is as effective as hospital based cardiovascular rehabilitation on a number of psychological, behavioural, biological, service and cost outcomes.[52] The Heart Manual included in a review of home-based and recently developed telehealth

			programmes, was identified as an effective alternative to hospital-based programmes.[53]
3.	What	**Materials:** Describe any physical or informational materials used in the intervention, including those provided to participants or used in intervention delivery or the training of intervention providers. Provide information on where the materials can be accessed (such as online appendix, URL)	The Heart Manual intervention package is written in simple language and laid out in **four, easy-to-follow parts**:<br>**Part 1: Your Heart Condition: The Facts.** This part contains important information for the patient to read during the initial phase of their recovery.<br>**Part 2: The Weekly Programme**: This part consists of six weekly sections, each containing important information to aid the patient's recovery, a graded exercise programme, and a focus each week on a lifestyle risk factor.<br>**Part 3: information and advice to help you out after (post myocardial infarction and revascularization):** This part contains important information about the patient's recovery, medication, and other significant issues relating to their condition such as mental health and well-being, hospital investigations, and treatments.<br>**Par 4: Cardiac risk factors and their management (Facts and advice to help you further prevention and control):** this part contains important information about how to prevent and control of heart disease risk factors.
4.		**Procedures:** Describe each of the procedures, activities, and/or processes used in the intervention, including any enabling or support activities	The key components and activities for a comprehensive cardiac rehabilitation programme are included in the Heart Manual supported by a specially trained physiotherapist, nurses and medical technologist:<br>➢ An exercise programme<br>➢ Lifestyle and risk factor education<br>➢ Advice about safe and unsafe activities including pacing<br>➢ The common psychological responses and their management<br>➢ Stress management and relaxation Information about frequently prescribed medications

			➢ Event and intervention-specific information, investigations, and treatments.<br>Patients should be encouraged to use the Heart Manual daily to help identify, plan, and monitor weekly targets and pacing activities. Psychological support and health behavior change activities are imbedded throughout the manual.
5.	Who provided	For each category of intervention provider (such as physiotherapist, psychologist, or nursing assistant), describe their expertise, background, and any specific training given	Patients should be assessed by a registered practitioner (nurse or AHP) as suitable to commence the programme. Non-registered/ non-regulated health care workers such as physiotherapists or health care support workers may be trained in the resources and deliver aspects of the programme under the supervision of a registered practitioner according to each individual's clinical competence.
6.	How provided	Describe the modes of delivery (such as face to face or by some other mechanism, such as internet or telephone) of the intervention and whether it was provided individually or in a group	**In hospital:** The 'Heart Manual' was first given in the Hospital through face-to-face communication. Initial in-hospital approach for recruitment-referral made /cardiac rehab (CR) team visit.<br>**Other form**-CR practitioner picks up referrals and approaches to make first contact with the patients by physical or phone for initial assessment confirmation and then send a 'Heart Manual' to the patient.<br>**Consultation format:** Formal assessment and consultation, information given/objective settings in clinic/ home/ or by phone/video call.<br>**Follow-up:** Phone follow-up, e.g., week 2/4. Tailored lifestyle content, objective, and behavior change by Phone/Video call.<br>**Final consultation check:** Understanding, long-term plan, emergency information by clinic/ home/ or by phone/video call.

7.	Where	Describe the type(s) of location(s) where the intervention occurred, including any necessary infrastructure or relevant features.	Its flexibility:<br>1. In hospital<br>2. Clinic<br>3. Home or alternative/suitable venue-e.g. community centre/hall<br>4. Phone call<br>5. Video call
8.	When and how much	Describe the number of times the intervention was delivered and over what period of time including the number of sessions, their schedule, and their duration, intensity, or dose	Initial assessment consultation will take about 1 hour.<br>➢ Subsequent contacts are likely to average about 30-45 mins.<br>➢ **Recovery program:** Following 6-week recovery programme:<br>**Week-1 Program:**<br>▪ Answer some more questions about your condition and why you feel the way you do.<br>▪ Introduce you to the Exercise and Relaxation Plans which will play an important part in your recovery and afterwards.<br>▪ Look at some of the things which might be worrying you, and show you how you can fight back.<br>▪ If you live with someone, get them to read this section - it can help them as much as it helps you.<br>**Week-2 program:**<br>▪ Try to carry on with the Exercise/Activity Plan.<br>▪ Don't forget to fill in your walking, exercise and activity record sheets. If you do this, you'll be able to look back<br>▪ at them in a few weeks and see how much you have improved.<br>▪ You will keep listening to the relaxation audio and get some tips about what to do if you are worried.<br>▪ You will learn about your coronary artery disease and the risk factors that contributed to it.<br>▪ You will find out what happened to John McKay (case study).

			▪ You will read about the biggest risk factor of all - smoking.<br>**Week-3 program:**<br>▪ Keep on building up your Exercise/Activity Plan. If you didn't start regular walking last week, try to do so now, as advised. If you did start regular walking, you should be building up the distance.<br>▪ Don't forget to fill in your walking, exercise and activity record sheets.<br>▪ Try to keep listening to the relaxation audio and get some information about stress and how to control it<br>▪ This week's risk factor is diet. Most people find that healthy food is also tasty - try it!<br>**Week-4 program:**<br>▪ Keep building up your Exercise/Activity Plan. You should also be walking daily and increasing the frequency, speed and distance as advised.<br>▪ Remember to fill in your walking, exercise and activity record sheets.<br>▪ Keep listening to the relaxation audio.<br>▪ There’s more this week about stress. A stressful lifestyle can play a big part in building up risk factors so it’s helpful to learn ways to manage it.<br>▪ This week’s risk factor is being overweight – a major problem for some people. But getting control of your weight can have a big benefit in reducing the risk of further heart problems in the future.<br>**Week-5 program:**<br>▪ You'll keep building up your Exercise/Activity Plan.<br>▪ Remember to fill in your walking, exercise and activity record sheets.<br>▪ Keep listening to the relaxation audio.

			▪ You'll hear more about stress. If you are thinking more and more about diving back into a busy lifestyle, you'll find some very useful advice about speeding, overworking and driven behaviour.<br>▪ This week's risk factor is lack of exercise. If you have been following the Exercise/Activity Plan you should already be seeing benefits from regular exercise, but this section goes into a little more detail about why it's so important.<br>▪ There's a section about sex (if you're interested).<br>**Week 6 - the last week of the Manual program:**<br>▪ You'll keep building up your Exercise/Activity Plan.<br>▪ Remember to fill in your walking, exercise and activity record sheets.<br>▪ Keep listening to the relaxation audio.<br>▪ We'll be looking at hostility and anger.<br>▪ We will think about going back to work. You may wish to discuss this with your GP or facilitator.<br>▪ This week's risk factor is high blood pressure.<br>▪ We shall bring together the different aspects of the stress control programs<br>▪ We shall review what has happened over the last 6 weeks and provide a checklist for future actions.<br>➢ Contacts can be face to face at home or in clinic, by telephone or video call (first contact benefits from being face to face if service provision allows)<br>➢ Phone calls are useful for checking in on patients between longer consultations If visiting patients at home, allow time for travel, record keeping etc.
9.	Tailoring	If the intervention was planned to be personalised, titrated or adapted, then describe what, why, when, and how	Be flexible:<br>➢ shorter, more frequent consultations may be useful for some patients<br>➢ some patients will need minimal input, shorter and/ or fewer sessions
