# Supplementary material for: Assessment of the Bangla Heart Manual in patients with coronary heart disease and their caregivers in Bangladesh: a feasibility study
Source: BMJ Open. 2026 Mar 30;16(3):e102350. doi: 10.1136/bmjopen-2025-102350 (PMC13052692; doi:10.1136/bmjopen-2025-102350)
Supplement: online supplemental file 4 [file bmjopen-16-3-s004.pdf]

eTable 2: Feasibility and acceptability outcome measures criteria.

Outcomes	Assessment measure	Success criteria	Time point
Feasibility	Number of eligible patients, number of patients screened, number of patients invited to take part, actual number of participants who consent to take part		After end of 6 weeks home based cardiac rehabilitation programme
Recruitment	Number of consented participants relative to the total number of eligible patients approached for consent	Recruitment rate of ≥30%	During study period
Retention	Number of participants who complete the final study assessment relative to the total number of participants enrolled for pilot trial.	Retention rate of ≥80%	During study period
Adherence	Percentage of completion of the Bangla Heart Manual intervention components according to training logs.	Intervention adherence of ≥75%	During study period
Adverse events	Adverse events reported during 6 weeks (walking, exercise and daily activities time) and weekly check-in sessions	No study-related adverse events	During (6 weeks follow-up) study period
**Acceptability outcome:** .	Participating patients and their caregivers will include a short questionnaire at a 6-week follow-up. This will also be supported by qualitative interviews with a subgroup of patients		After end of 6 weeks home based cardiac rehabilitation programme
**Satisfaction**	Patients and their caregivers (by using (0 to 5 Likert scale)	≥80% reporting above-average or excellent satisfaction on a Likert scale	After end of 6 weeks home based cardiac rehabilitation programme
