# Supplementary material for: Assessment of the Bangla Heart Manual in patients with coronary heart disease and their caregivers in Bangladesh: a feasibility study
Source: BMJ Open. 2026 Mar 30;16(3):e102350. doi: 10.1136/bmjopen-2025-102350 (PMC13052692; doi:10.1136/bmjopen-2025-102350)
Supplement: online supplemental file 5 [file bmjopen-16-3-s005.pdf]

eTable 3: Secondary outcome measures by time point.

**Secondary outcomes**	**Assessment measure**	**Time points**
Sociodemographic characteristics	Descriptive	At Baseline
Clinical	Descriptive	At Baseline
Exercise and functional capacity	Incremental Shuttle Walk Test (ISWT)	Baseline and at the last follow-up time point
**Hospitalisations**:	number, reason and duration	During 6 weeks follow-up period
**Mortality**	reason and how many days later after procedure.	During 6 weeks follow-up period
**Patient reported outcome**		
Disease-specific Health-Related Quality of Life, (HRQoL)	Measured using the HeartQoL Bangla version questionnaire.	Baseline and at the last follow-up time point
EQ-5D-5L	Generic quality of life five-dimension EuroQol (EQ-5D-5L) Scale	Baseline and at the last follow-up time point
HADS	Psychological well-being: Hospital Anxiety and Depression Scale (HADS).	Baseline and at the last follow-up time point
