# Supplementary material for: Assessment of the Bangla Heart Manual in patients with coronary heart disease and their caregivers in Bangladesh: a feasibility study
Source: BMJ Open. 2026 Mar 30;16(3):e102350. doi: 10.1136/bmjopen-2025-102350 (PMC13052692; doi:10.1136/bmjopen-2025-102350)
Supplement: online supplemental file 6 [file bmjopen-16-3-s006.pdf]

ISRCTN15454620 https://doi.org/10.1186/ISRCTN15454620

Evaluates the feasibility and acceptability of the Bangla Heart Manual, a home-based cardiac rehabilitation program, for patients with coronary heart disease (CHD) and their caregivers in Bangladesh

Submission date	Recruitment status	[ ] Prospectively registered
19/03/2025	No longer recruiting	[X] Protocol
Registration date	Overall study status	[ ] Statistical analysis plan
23/07/2025	Completed	[ ] Results
Last Edited	Condition category	[ ] Individual participant data
23/07/2025	Circulatory System	[X] Record updated in last year

Plain English summary of protocol
Background and study aims
Coronary heart disease (CHD) is a leading cause of death and disability worldwide, especially in low- and middle-income countries (LMICs) like Bangladesh. Cardiac rehabilitation (CR) is a proven way to help people with heart disease recover, but it is underused in LMICs due to barriers like cost, travel, and lack of facilities. This study aims to assess the feasibility and acceptability of an adapted Bangla Heart Manual as an intervention for CHD patients and their caregivers. The study also explored whether the program improved exercise capacity and quality of life.

Who can participate?
Patients aged 18 years old and over with CHD (e.g., previous heart attack or angina) who were admitted to Ibrahim Cardiac Hospital & Research Institute in Dhaka, Bangladesh, for heart surgery or angioplasty between June and July 2024.

What does the study involve?
Participants received the Bangla Heart Manual, a 6-week home-based CR program. It included exercise training, education on healthy living, and psychological support. Patients were introduced to the program by a physiotherapist in the hospital and received weekly phone calls to track their progress. Data on exercise capacity, quality of life, and psychological well-being were collected before and after the program. Patients and caregivers were also interviewed about their experiences.

What are the possible benefits and risks of participating?
Benefits included improved exercise capacity, better quality of life, and learning how to manage heart disease. Risks were minimal, but two participants died during the study due to unrelated medical reasons. No risks were linked to the CR program itself.

Where is the study run from?
The Ibrahim Cardiac Hospital & Research Institute in Dhaka, Bangladesh.

When does the study start and how long will it run?
May 2024 to September 2024

Who is funding the study?
The study was funded by the University of Glasgow, UK, as part of a PhD research project.

Who is the main contact?
Md. Jamal Uddin, a PhD research student at the University of Glasgow, 2780885U@student.gla.ac.uk or uddinj83@gmail.com

Contact information

Type(s)
Public, Scientific, Principal investigator

Contact name
Mr Md. Jamal Uddin

ORCID ID
https://orcid.org/0000-0001-5964-6381

Contact details
PhD research student
MRC & CSO Social and Public Health Sciences Unit
College of Medical, Veterinary and Life Sciences
University of Glasgow
Senior Chest Physiotherapist
Department of Cardiac Surgery,
Ibrahim Cardiac Hospital & Research Institute, Shahbag, Dhaka, Bangladesh
Dhaka
Bangladesh
Dhaka-1000
+88-01824630856
2780885U@student.gla.ac.uk

Additional identifiers

Clinical Trials Information System (CTIS)
Nil known

ClinicalTrials.gov (NCT)
Nil known

Protocol serial number
Nil known

Study information

Scientific Title
Assessment of the Bangla Heart manual in patients with coronary heart disease and their caregivers in Bangladesh: a feasibility study

Study objectives
Study hypothesis was that the adapted Bangla Heart Manual was a feasible and acceptable home-based cardiac rehabilitation programme for people with CHD and their caregivers in Bangladesh.

Ethics approval required
Ethics approval required

Ethics approval(s)
approved 16/05/2024, Ethical Review Committee, Ibrahim Cardiac Hospital & Research Institute (Ibrahim Cardiac Hospital & Research Institute, 122, Kazi nazrul Islam Avenue, Shahbag, Dhaka, Dhaka-1000, Bangladesh; +88-02-41060451-58; info@ibrahimcardiac.org.bd), ref: Ref-ICHRI /Research/ERC/2024/05

Study design
Mixed-methods single-arm design with pre-post assessment of patient outcomes

Primary study design
Interventional

Study type(s)
Quality of life, Treatment

Health condition(s) or problem(s) studied
CHD diagnosis defined as either a previous myocardial infarction (MI) or angina pectoris (AP) who were admitted in a hospital for an intended revascularisation intervention (angioplasty and or coronary artery bypass graft (CABG) procedures)

Interventions
The study employed a mixed-methods single-arm design with pre-post assessment of patient outcomes and qualitative interviews with healthcare staff involved in the intervention delivery, patients and caregivers. Patients were recruited from the Ibrahim Cardiac Hospital & Research Institute based in Dhaka, Bangladesh.

The Bangla Heart Manual is a home-based, health professional-facilitated, 6-week CR programme supporting self-care in patients with CHD. In brief, the Bangla Heart Manual includes the key elements of a comprehensive CR programme i.e. exercise training, psychological support, and education around self-management including medial therapy and healthy lifestyle.

Patients were introduced to the program by a physiotherapist in the hospital and received weekly phone calls to track their progress. Data on exercise capacity, quality of life, and psychological well-being were collected before and after the program. Patients and caregivers were also interviewed about their experiences.

Intervention Type
Behavioural

Primary outcome(s)
Feasibility:
1. Recruitment: the number of consented participants relative to the total number of eligible patients approached for consent, measured using screening logs at 1.5 months
2. Retention: the number of participants who complete the final study assessment relative to the total number of participants enrolled for the pilot trial, measured using study logs at 3-months follow-up
3. Adherence: the percentage completion of the Bangla Heart Manual intervention components measured using training logs at 6 weeks follow-up
4. Adverse events: adverse events (walking, exercise and daily activities time) measured using self-reporting and weekly check-in sessions at 6 weeks of follow-up

Acceptability:
1. Acceptability: participating patients and their caregivers' acceptability of the intervention and its delivery measured using a questionnaire and qualitative interviews at the end of the 6-week home-based cardiac rehabilitation programme
2. Satisfaction: the satisfaction of patients and their caregivers measured using a questionnaire (0 to 5 Likert scale) at the end of the 6-week home-based cardiac rehabilitation programme

Key secondary outcome(s))
1. Sociodemographic characteristics: details of patient's age, sex etc measured using self-reporting and medical records at baseline
2. Clinical characteristics: details of patient's clinical status measured using medical records at baseline
3. Exercise and functional capacity measured using Incremental Shuttle Walk Test (ISWT) at baseline and 6-weeks follow-up
4. Hospitalisation measured using self-reporting at 6 weeks follow-up period
5. Mortality measured using medical record at 6 weeks follow-up period
6. Patient reported outcomes will be measured using the Disease-specific Health-Related Quality of Life, (HRQoL), HeartQoL Bangla version questionnaire, Generic specific Health-Related Quality of Life, (HRQoL) and the Generic quality of life five-dimension EuroQol (EQ-5D-5L) Scale at baseline and 6-weeks follow-up
7. Psychological well-being measured using the Hospital Anxiety and Depression Scale (HADS) at baseline and 6-weeks follow-up

Completion date
30/09/2024

Eligibility

Key inclusion criteria
1. Consecutive patients aged ≥18 years
2. A CHD diagnosis, defined as either a previous myocardial infarction (MI) or angina pectoris (AP)
3. Admitted to a hospital for an intended revascularisation intervention (angioplasty and or coronary artery bypass graft (CABG) procedures) between June and July 2024
4. Deemed suitable for CR participation by the clinical team

Participant type(s)
Patient, Carer

Healthy volunteers allowed
No

Age group
Mixed

Lower age limit
18 years

Upper age limit
80 years

Sex
All

Total final enrolment
33

Key exclusion criteria
1. Underwent emergency revascularization
2. Had undertaken CR within the last 12 months
3. Had contraindications to exercise testing or exercise training as part of their documented medical history
4. Were unwilling or unable to travel to the clinical site for their research assessments

Date of first enrolment
01/06/2024

Date of final enrolment
15/07/2024

Locations

Countries of recruitment
Bangladesh

Study participating centre
Ibrahim Cardiac Hospital & Research Institute
122, Kazi Nazrul Islam Avenue, Shahbag
Dhaka
Bangladesh
Dhaka-1000

Sponsor information

Organisation
Ibrahim Cardiac Hospital & Research Institute

ROR
https://ror.org/008971d44

Funder(s)

Funder type
University/education

Funder Name
University of Glasgow

Alternative Name(s)

Funding Body Type
Private sector organisation

Funding Body Subtype
Universities (academic only)

Location
United Kingdom

Results and Publications

Individual participant data (IPD) sharing plan
The datasets generated during and/or analysed during the current study are/will be available upon request from the corresponding author, Md. Jamal Uddin, 2780885U@student.gla.ac.uk or uddinj83@gmail.com

IPD sharing plan summary
Available on request

Study outputs

Output type	Details	Date created	Date added	Peer reviewed?	Patient-facing?
Protocol (other)		09/11/2024	20/03/2025	No	No
