# Supplementary material for: Assessment of the Bangla Heart Manual in patients with coronary heart disease and their caregivers in Bangladesh: a feasibility study
Source: BMJ Open. 2026 Mar 30;16(3):e102350. doi: 10.1136/bmjopen-2025-102350 (PMC13052692; doi:10.1136/bmjopen-2025-102350)
Supplement: Supplementary file 7 [file bmjopen-2025-102350.reviewer_comments.pdf]

PEER REVIEW HISTORY

BMJ Open publishes all reviews undertaken for accepted manuscripts. Reviewers are asked to complete a checklist review form and are provided with free text boxes to elaborate on their assessment. These free text comments are reproduced below.

ARTICLE DETAILS

Title (Provisional)

ASSESSMENT OF THE BANGLA HEART MANUAL IN PATIENTS WITH CORONARY HEART DISEASE AND THEIR CAREGIVERS IN BANGLADESH: A FEASIBILITY STUDY

Authors

Uddin, Jamal; Faruque, Mithila; Mashreky, Saidur Rahman; Siddiqueea , Yousra; Chowdhury, Meshkat A.; Karim, Rezaul; Khaled, Md Fakhrul Islam; Taylor, Louise; Dalal, Hasnain M; Taylor, Rod

VERSION 1 - REVIEW

**Reviewer**	**1**
**Name**	**Dewi, Ayu Putu Madri**
**Affiliation**	**Amsterdam UMC Location AMC, Epidemiology and Data Science**
**Date**	**16-Apr-2025**
**COI**	**None**

Manuscript Title: Assessment of The Bangla Heart Manual in Patients With Coronary Heart Disease and Their Caregivers in Bangladesh: A Feasibility Study

Dear authors,

I am an Open Science researcher, who has checked your manuscript for its adherence to a number of Open Science practices.

Below you find the checklist that I used to assess your manuscript for adherence to a number of these practices.

Based on this assessment, I would suggest the following refinements/additions:

1. Availability of a protocol: OK.

2. Pre-registration: OK.

3. Open Materials: OK

4. Open Data: Given the confidentiality of the data, please specify whether de-identified, metadata, or a subset of the data can still be made publicly available.

Note, if this paper is accepted, you will be required to add a data availability statement and may be required to share data by BMJ/BMJ Open Policy. For more information, please see: https://authors.bmj.com/policies/data-sharing/ ; https://bmjopen.bmj.com/pages/authors

5. Open Code: Please provide (a link to) the code used for the analyses in this study.

Note that for papers accepted at The BMJ you will be required to provide a supplementary file with the code if this paper is accepted. For more information, please see Data sharing section on the website: https://authors.bmj.com/policies/data-sharing/ (The BMJ); https://bmjopen.bmj.com/pages/authors (BMJ Open)

6. Citations: Please provide citations for your materials/data/codes.

7. Software and tools used are described: Please explicitly mention which software and tools were used in your study, including references to versions.

8. Open access publication: OK.

9. Reporting checklist provided: OK

10. Preprint: Please state explicitly whether and where the preprint of this study can be found.

Reviewer 2

Name Zheng, Zhe

Affiliation Fuwai Hospital, National Center for Cardiovascular Diseases, Chinese Academy of Medical Sciences and Peking Union Medical College, National Clinical Research Center of Cardiovascular Diseases, State Key Laboratory of Cardiovascular Disease

Date 19-May-2025

COI None

This paper introduces the implementation of a post-revascularization cardiac rehabilitation program in the authors’ country, providing a useful reference for similar work in the region. However, the study has several issues that need further improvement:

The surgical procedures among enrolled patients vary considerably. There are significant differences in postoperative rehabilitation between CABG and PCI patients, and it is not advisable to apply a single rehabilitation program to both groups.

The sample size is small, and the enrollment rate is low. Although 72 patients were screened, only 33 were ultimately included. The reasons for excluding the remaining patients should

be clarified, as the low enrollment rate may affect the feasibility of the rehabilitation program.

While the study attempts to provide insights into postoperative rehabilitation for coronary heart disease patients in low- and middle-income countries, it does not clearly differentiate itself from previous studies conducted in high-income countries. As a result, the study's originality is limited.

**Reviewer**	**3**
**Name**	**Kelson, Zoe**
**Affiliation**	**University of Exeter, Mathematics**
**Date**	**17-Dec-2025**
**COI**	**None**

This study aims to assess the feasibility and acceptability of the home-based Bangla Heart Manual of CR programme for people with CHD following revascularization living in Bangladesh.

Reviewer comments:

"The study is reported in accordance with the Consolidated Standards of Reporting Trials (CONSORT) extension for randomised pilot and feasibility trials"

Can a copy of the associated reporting checklist please be provided?

"Tertiary level cardiac hospital in Dhaka city...

The study involved 33 CHD patients admitted for revascularization (CABG or PCI) between June and July 2024, selected from 72 screened." [Abstract]

and

"Patients were recruited from the Ibrahim Cardiac Hospital & Research Institute based in Dhaka, Bangladesh"

and

"A total of 33 CHD patients were recruited, that included 29 males (88%) with a mean age of 55 (SD: 7.7) years, and underwent either CABG (70%) and PCI (30%) (see table 1). "

Can the authors please comment on whether they deem the included cohort to be representative?

In particular, the sample appears to be male dominated?

"Baseline data included baseline medical and demographic characteristics and at both baseline and 3-months follow up: exercise capacity a range of participant reported questionnaires (see below) and patient and caregiver satisfaction. "

Can the authors please confirm if there was any missing data?

Can the potential for self-reporting bias please be acknowledged?

"To achieve the objectives of this study and based on the recommendation of Treweek et al 30 for pilot and feasibility trials of minimum of 10 to 15 participants, the study planned to recruit a total of 30 patients over a 2-months period allowing for loss to follow-up of up to 20% at 3 months follow up"

The basis for the sample size assessment has been suitably communicated by the authors.

"Pre-post differences in outcomes are presented as means and 95% confidential intervals (CIs). Given the exploratory nature of the study, the focus of outcome interpretation was around 95% CIs rather than P-values."

Can statistical testing please be conducted, albeit p-values can be interpreted as exploratory only?

Can the authors please undertake a paired data analysis?

Can potential confounding (e.g. time dependent variables) please be adjusted for in the analyses?

"Interview data were interpreted by two qualitative researchers by developing a set of codes that were applied to the transcripts to identify themes. "

Can the authors please provide a citation for the type of thematic analysis applied?

"Table 4: Secondary outcomes: Exercise capacity (ISWT) at baseline and 3-months follow up "

and

"Table 5: Secondary outcomes: HeartQoL physical and emotional subscale, HADS and EQ-5D-5L at baseline and 3-months follow up"

Can the authors please clarify what statistical method(s) were applied to generate the p-values reported in these tables?

"Baseline assessments were conducted before revascularization, making it unclear whether improvements in exercise capacity and patient-reported outcomes were due to the CR intervention, revascularization, or both. ➢ Lack of a control group, small sample size, and short follow-up period restrict the study's generalizability and depth of findings. ➢ The study did not formally assess the efficacy of the Bangla Heart Manual CR intervention, focusing instead on feasibility and acceptability." [Strengths and Limitations of this Study]

and

"Whilst not formally powered for efficacy..."

Can the Discussion of the study limitations please be expanded on?

VERSION 1 - AUTHOR RESPONSE

Reviewer's comments	Response	Manuscript revision
**Reviewer 1:**		
1. Availability of a protocol: OK.	We thank the reviewer for confirmation .	No change required
2. Pre-registration: OK.	We thank the reviewer for this confirmation.	No change required
3. Open Materials: OK	We thank the reviewer for this confirmation.	No change required
4. Open Data: Given the confidentiality of the data, please specify whether de-identified, metadata, or a subset of the data can still be made publicly available.<br>Note, if this paper is accepted, you will be required to add a data availability statement and may be required to share data by BMJ/BMJ Open Policy. For more information, please see: https://authors.bmj.com/policies/data-sharing/ ; https://bmjopen.bmj.com/pages/authors	Data sharing information statement has been added	See blind title page, Page 2.

5. Open Code: Please provide (a link to) the code used for the analyses in this study.<br>Note that for papers accepted at The BMJ you will be required to provide a supplementary file with the code if this paper is accepted. For more information, please see Data sharing section on the website: https://authors.bmj.com/policies/data-sharing/ (The BMJ); https://bmjopen.bmj.com/pages/authors (BMJ Open)	Details of analysis have been added to methods. Provision of open code for analysis not applicable in this case.	See methods section for analysis.
6. Citations: Please provide citations for your materials/data/codes.	Reference added.	Reference added : Uddin J., Faruque M., Mashreky S. R., *et al.* Adaptation of an evidence-based home cardiac rehabilitation programme for people with coronary heart disease in Bangladesh. *BMC Health Serv Res* (2026). Accepted for publication https://doi.org/**10.1186/s12913-026-14056-6**
7. Software and tools used are described: Please explicitly mention which software and tools were used in your study, including references to versions.	Thank you Details added to analysis section.	Added to the Methods and Analysis section: " Quantitative data were analysis using SPSS V.23.0”
8. Open access publication: OK.	We thank the reviewer for this confirmation.	No change required

9. Reporting checklist provided: OK	We thank the reviewer for this confirmation.	
10. Preprint: Please state explicitly whether and where the preprint of this study can be found.	We confirm a preprint of this study has not been published.	No change required
**Reviewer: 2**		
1. This paper introduces the implementation of a post-revascularization cardiac rehabilitation program in the authors' country, providing a useful reference for similar work in the region. However, the study has several issues that need further improvement.	Thank you.	No change required
2. The surgical procedures among enrolled patients vary considerably. There are significant differences in postoperative rehabilitation between CABG and PCI patients, and it is not advisable to apply a single rehabilitation program to both groups.	We agree with the reviewer that rehabilitation needs can differ between CABG and PCI patients. The Bangla Heart Manual was specifically adapted to address this. We have clarified this in the "Bangla Heart Manual intervention" subsecti on of the methods.	Main manuscript, Page 4, Methods section, subsection of *Bangla Heart Manual intervention*: "Within the Bangla Heart Manual intervention, distinct exercise programmes were incorporated for patients following coronary artery bypass grafting (CABG) and percutaneous coronary intervention (PCI). Rehabilitation professionals including physicians, physiotherapists, nurses, and dietitians—were trained to deliver these tailored components appropriately."
3. The sample size is small, and the enrollment rate is low. Although 72	A sample size (>30) is appropriate for a	Page 7, Results section: "33 (46%) patients were

patients were screened, only 33 were ultimately included. The reasons for excluding the remaining patients should be clarified, as the low enrollment rate may affect the feasibility of the rehabilitation program.	feasibility study. Details of exclusions have been added, .	recruited out of 72 patients screened over a 2-month period... This represents 100% consent rate among eligible patients (33/33). The 39 patients screened but not recruited were excluded based on pre-defined eligibility criteria."
4. While the study attempts to provide insights into postoperative rehabilitation for coronary heart disease patients in low- and middle-income countries, it does not clearly differentiate itself from previous studies conducted in high-income countries. As a result, the study's originality is limited.	We respectfully n the disagree with the reviewer and contend this study is original. WE have added a statement to this effect in the discussion section	Page 9, Discussion section: "This study provides proof-of-concept for a culturally adapted, home-based CR model delivered Added in to the manuscript: Yes – in Introduction (paragraph detailing adaptation process) and Discussion (section on implications for practice/research).
**Reviewer 3:**		
1. "The study is reported in accordance with the Consolidated Standards of Reporting Trials (CONSORT) extension for randomised pilot and feasibility trials" Can a copy of the associated reporting checklist please be provided?	The completed CONSORT extension for pilot and feasibility trials checklist has been uploaded as a supplementary file.	Supplementary file uploaded separately (see Supplementary File 1, Figure 2).
2. "Tertiary level cardiac hospital in Dhaka city... The study involved 33 CHD	We acknowledge that the study cohort was not potentially	Added to the discussion and Strengths & limitations section: "Furthermore, the

patients admitted for revascularization (CABG or PCI) between June and July 2024, selected from 72 screened." [Abstract]<br>and<br>"Patients were recruited from the Ibrahim Cardiac Hospital & Research Institute based in Dhaka, Bangladesh"<br>and<br>"A total of 33 CHD patients were recruited, that included 29 males (88%) with a mean age of 55 (SD: 7.7) years, and underwent either CABG (70%) and PCI (30%) (see table 1). "<br>Can the authors please comment on whether they deem the included cohort to be representative?	representative of the broader Bangladeshi CHD population.	single-center recruitment from an urban tertiary hospital and the predominantly male sample limit the generalizability of our feasibility findings to the wider Bangladeshi population, including women and those in rural or primary care settings."
3. In particular, the sample appears to be male dominated?	Thank you for this point	This issue has been added to the limitations in the Discussion section.
4. "Baseline data included baseline medical and demographic characteristics and at both baseline and 3-months follow up: exercise capacity a range of participant reported questionnaires (see below) and patient and caregiver satisfaction. "<br>Can the authors please confirm if there was any missing data?	There was minimal missing data. Complete outcome data at 3-month follow-up was obtained from 30/33 participants (91%). The three missing cases were due to two deaths and one	Clarified in the Results section under "Primary Feasibility outcomes": "30 of 33 (91%) participants provided complete outcome data at 3-months follow-up. The three missing cases were due to two deaths and one dropout, none related to the intervention.

	dropout, none related to the intervention.	
5. Can the potential for self-reporting bias please be acknowledged?	Thank you for this important point.	Added to the discussion and Strengths & Limitations section: "Additionally, the reliance on self-reported measures for HRQoL and psychological well-being may be subject to reporting bias."
6. "To achieve the objectives of this study and based on the recommendation of Treweek et al 30 for pilot and feasibility trials of minimum of 10 to 15 participants, the study planned to recruit a total of 30 patients over a 2-months period allowing for loss to follow-up of up to 20% at 3 months follow up"<br>The basis for the sample size assessment has been suitably communicated by the authors.	Thank you.	No change needed
7. "Pre-post differences in outcomes are presented as means and 95% confidential intervals (CIs). Given the exploratory nature of the study, the focus of outcome interpretation was around 95% CIs rather than P-values."<br>Can statistical testing please be conducted, albeit p-values can be interpreted as exploratory only?	We have conducted paired-sample t-tests (for continuous variables) and have included p-values alongside the 95% CIs, with the clear caveat that these are exploratory due to the feasibility study design.	Methods: Updated to: "Pre-post differences in outcomes are presented as means and 95% confidence intervals (CIs). Exploratory paired-sample t-tests were conducted for continuous variables, with p-values reported for descriptive purposes only." Results (Tables 4 & 5): p-values from paired t-tests have been added.

8. Can the authors please undertake a paired data analysis?	As above, paired data analyses (paired-sample t-tests) has been conducted.	See above
9. Can potential confounding (e.g. time dependent variables) please be adjusted for in the analyses?	In this single-arm feasibility study with a small sample, the primary aim was not to establish efficacy or adjust for confounders, which would require a controlled trial. important confounders, which is acknowledged as a key limitation.	As above, discussion and & Limitations and Discussion include this limitation,
10. "Interview data were interpreted by two qualitative researchers by developing a set of codes that were applied to the transcripts to identify themes. " Can the authors please provide a citation for the type of thematic analysis applied?	We thank the Reviewer for highlighting this important point .	Added to the Methods: "Interview data (qualitative data were analyzed using thematic analysis, as described by Braun and Clarke (2006), by two qualitative researchers who developed codes and identified themes." Reference added: Braun, V., & Clarke, V. (2006). Using thematic analysis in psychology. Qualitative Research in Psychology, 3(2), 77-101.
11. "Table 4: Secondary outcomes: Exercise capacity (ISWT) at baseline and 3-months follow	The p-values in the revised tables are derived from	Table 4 & 5 footnotes: "p-values derived from

up "<br>and<br>"Table 5: Secondary outcomes: HeartQoL physical and emotional subscale, HADS and EQ-5D-5L at baseline and 3-months follow up"<br>Can the authors please clarify what statistical method(s) were applied to generate the p-values reported in these tables?	exploratory paired-sample t-tests, as requested.	exploratory paired-sample t-tests."
12. "Baseline assessments were conducted before revascularization, making it unclear whether improvements in exercise capacity and patient-reported outcomes were due to the CR intervention, revascularization, or both. ➢ Lack of a control group, small sample size, and short follow-up period restrict the study's generalizability and depth of findings. ➢ The study did not formally assess the efficacy of the Bangla Heart Manual CR intervention, focusing instead on feasibility and acceptability."<br>[Strengths and Limitations of this Study]<br>and<br>"Whilst not formally powered for efficacy..."<br>Can the Discussion of the study limitations please be expanded on?	As above, the discussion section has been expanded to consider the study key limitations.	As above.
